# Characterization of the complete chloroplast genome of the Hongkong kumquat (*Fortunella hindsii* Swingle)

**DOI:** 10.1080/23802359.2019.1642166

**Published:** 2019-07-17

**Authors:** Shi-Rong Xu, Yong-Yan Zhang, Fan Liu, Na Tian, Dong-Ming Pan, Xue-Jun Bei, Chun-Zhen Cheng

**Affiliations:** a College of Horticulture, Fujian Agriculture and Forestry University, Fuzhou, P. R. China;; b College of Biology and Pharmacy, Yulin Normal University, Yulin, P. R. China

**Keywords:** *Fortunella hindsii*, Hongkong kumquat, citrus species, chloroplast genome, phylogenetic relationship analysis

## Abstract

Hongkong qumquat (*Fortunella hindsii* Swingle) is a wild citrus species native to China. In this study, we firstly reporteded its complete chloroplast genome using BGISEQ-500 sequencing. The chloroplast genome is 160,145 bp in size, containing a large single copy region (87,467 bp), a small single copy region (18,730 bp), and a pair of IR regions (26,974 bp). The chloroplast genome contains 112 unique genes, including 79 protein-coding genes, 29 tRNAs, and 4 rRNAs. Phylogenetic maximum-likelihood analysis indicated that *F. hindsii* is closely related to *Citrus* species. The complete chloroplast genome would be subsequently used for citrus species researches.

Hongkong kumquat (*Fortunella hindsii* Swingle), belonging to the subfamily Aurantioideae of the Rutaceae, is a wild citrus species widely distributed in low-altitude mountainous areas of Fujian, Jiangxi, Guangdong, Guangxi and some other southern provinces of China. It is native to South China, people there usually apply its fruits as condiments, use its dried roots and leaves as Chinese folk-medicine, and made the *F. hindsii* plant into miniascape (Zhu et al. 2019). Moreover, due to its high callus induction ratio from *F. hindsii* explants and short juvenility characteristics, it was recommended to be used as model species for citrus studies (Zhang et al. 2009; Zhu et al. 2019). In this study, we obtained the complete chloroplast genome of *F. hindsii* and explored its phylogenetic relationship with several citrus species, which would provide valuable genetic information for the plants of Rutaceae and can be subsequently used for researches of citrus species.

The specimen of *F. hindsii* was isolated from Fengping village, Malu Town, Dongping city, Guangxi province, China (21°40′19.24″N; 108°0′16.74″E) and samples were deposited at Fujian Agriculture and Forestry University. The leaf genomic DNA was extracted using Plant Genomic DNA Kit (TIANGEN Biotech, Beijing, China) and stored at the Fujian Agriculture and Forestry University (No. FAFUSJG01). The whole genomic DNA sequencing was performed on the BGISEQ-500 Sequencing platform to generate 125 bp pair-end reads (BIG, Shenzhen, China). Approximately 1.3 Gbp high-quality reads were obtained, and clean reads were aligned to chloroplast genomes of *Citrus reticulata* (KY596676.1), *C. limon* (KY085897.1), *C. depressa* (LC147381.1), *C. sinensis* (DQ864733.1), *C. platymamma* (KR259987.1) and *C. aurantiifolia* (KJ865401.1), and then assembled into contigs using CLC Genomics Workbench v8.0 (CLC Bio, Aarhus, Denmark). DOGMA (Wyman et al. 2004) and Geneious (Kearse et al. 2012) were applied for chloroplast genome annotation. And the annotated chloroplast genome has been deposited in Genbank with the accession number MN073195.

The complete chloroplast genome of *F. hindsii* is 160,145 bp in size, containing a large single copy region of 87,467 bp, a small single copy region of 18,730 bp, and a pair of inverted repeat (IR) regions of 26,974 bp. The chloroplast contains 112 unique genes, including 79 protein-coding genes, 29 tRNA genes, and 4 rRNA genes. Most of them (90) occur as a single copy, but 10 protein-coding genes (i.e. *ycf1*, *ycf2*, ycf15, *rps7*, *rps12*, *rps19*, *rpl2*, *rpl22*, *rpl23*, and *ndhB*), 8 tRNA genes (i.e. *trnA-UGC*, trnG-GCC, *trnI-CAU, trnI-GAU*, *trnL-CAA*, *trnN-GUU*, *trnR-ACG*, and *trnV-GAC*) and all the 4 rRNA genes (*4.5S*, *5S*, *16S*, and *23S* rRNA) occur in double copies. The overall nucleotide composition of the chloroplast genome is 30.5% A, 31.1% T, 19.6% C, and 18.9% G, with the total GC content being 38.4%.

We constructed a maximum-likelihood phylogenetic tree using the complete chloroplast genomes of *F. hindsii* and 19 plant species from Rutaceae family with *Ailanthus altissima* as outgroup. The sequences were aligned using HomBlocks pipeline (Bi et al. 2018) and then RAxML-HPC2 on XSEDE version 8.2.10 (Stamatakis 2014) was used for the construction of a maximum-likelihood tree with branch support computed with 1000 bootstrap replicates. The result showed that *F. hindsii* is closely related to *Citrus* species (Figure 1). The complete chloroplast genome of *F. hindsii* would provide valuable genetic information for its genetic relationships with other plant species of Rutaceae and would be subsequently used for citrus species researches.

**Figure 1.**
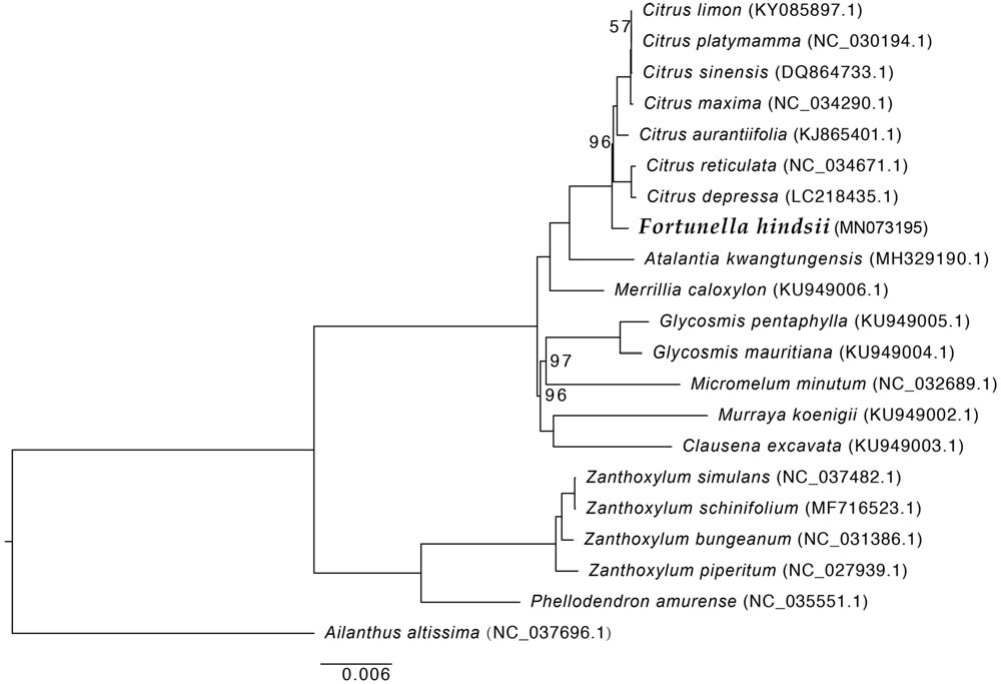
Maximum-likelihood tree based on the complete chloroplast genome sequences of *F. hindsii* and 19 plant species from the Rutaceae with *Ailanthus altissima* as outgroup. Numbers shown next to the nodes are bootstrap support values based on 1000 replicates, bootstrap values of 100 were omitted.
